# Porcine Intestinal Organoids: Overview of the State of the Art

**DOI:** 10.3390/v14051110

**Published:** 2022-05-21

**Authors:** Panpan Ma, Puxian Fang, Tianze Ren, Liurong Fang, Shaobo Xiao

**Affiliations:** 1 State Key Laboratory of Agricultural Microbiology, College of Veterinary Medicine, Huazhong Agricultural University, Wuhan 430070, China; panpma@163.com (P.M.); rtz199897@163.com (T.R.); fanglr@mail.hzau.edu.cn (L.F.); vet@mail.hzau.edu.cn (S.X.); 2 The Key Laboratory of Preventive Veterinary Medicine in Hubei Province, Cooperative Innovation Center for Sustainable Pig Production, Wuhan 430070, China

**Keywords:** porcine intestinal organoids, in vitro model, intestinal development, host–microbe interactions, drugs discovery

## Abstract

The intestinal tract is a crucial part of the body for growth and development, and its dysregulation can cause several diseases. The lack of appropriate in vitro models hampers the development of effective preventions and treatments against these intestinal tract diseases. Intestinal organoids are three-dimensional (3D) polarized structures composed of different types of cells capable of self-organization and self-renewal, resembling their organ of origin in architecture and function. Porcine intestinal organoids (PIOs) have been cultured and are used widely in agricultural, veterinary, and biomedical research. Based on the similarity of the genomic sequence, anatomic morphology, and drug metabolism with humans and the difficulty in obtaining healthy human tissue, PIOs are also considered ideal models relative to rodents. In this review, we summarize the current knowledge on PIOs, emphasizing their culturing, establishment and development, and applications in the study of host–microbe interactions, nutritional development, drug discovery, and gene editing potential.

## 1. Introduction

The intestinal tract is an essential digestive organ, playing important functions in the digestion, absorption, and metabolism of food, including the metabolism and absorption of vitamins [1], amino acids [2], and lipids [3]. The intestinal epithelium consists of cells located at the mucosal surface. These intestinal epithelial cells facilitate the digestion of food and nutrient absorption. The intestinal epithelial surface area is increased significantly by the formation of small intestinal villi and microvilli [4], making the intestinal epithelial surface more susceptible to external environmental stimuli, including food antigens, toxins, and microbial pathogens. Intestinal epithelial cells undergo self-renewal every 3 to 5 days to maintain tissue homeostasis and barrier function [5]; thus, the intestine is one of the most actively regenerated tissues. The intestinal epithelium consists of different cell types, including enterocytes, enteroendocrine cells, goblet cells, Paneth cells, and stem cells. Enterocytes are responsible for nutrient absorption [6], enteroendocrine cells regulate metabolism by secreting different hormones [6], goblet cells form a mucosal barrier by synthesizing and secreting mucins [7], and Paneth cells provide support to stem cells by secreting various factors and also secrete antimicrobial peptides for defense against pathogens [8]. These differentiated epithelial cells originate from intestinal stem cells located at the base of the crypt. The entire intestinal cell renewal process is facilitated by differentiation along the crypt to the villi through the activation of the Wnt/β-catenin signaling pathway [9], which is highly conserved in the animal kingdom [10].

Currently, laboratory animals are used predominantly to simulate the physiological and pathophysiological functions of the human intestinal tract. In comparison with other animal models, the porcine intestine is superior because of its anatomical and physiological similarities to humans [11]. Therefore, in-depth studies of the porcine intestinal epithelium play a crucial role in agricultural, veterinary, and biomedical research. Previous studies on porcine enteric disease were performed using immortalized cell lines of porcine or non-porcine origin, such as IPEC-J2 (porcine jejunal epithelial cells) [12], IPI-2I [13] (porcine ileal epithelial cells), and Vero (African green monkey kidney cells) [14]. However, cell immortalization is usually performed via viral infection, cell fusion, or oncogenes, which may affect the normal or intact biological function of cell lines [15]. Mimicking the biological process of a pathogen-induced immune response is difficult to achieve with a single cell line [16]. Moreover, immortalized cell lines have other defects. For example, Vero cells fail to produce type I interferon (IFN) because of an interferon deficiency when infected by a virus [17]. The clinical isolates of the porcine epidemic diarrhea virus (PEDV) do not usually replicate well in pig-derived cells, such as IPEC-J2 and IPI-2I cells [18]. Therefore, in vitro studies require appropriate models to reproduce the complexity of the intestinal epithelium, which is essential for improving the reliability of results.

The emergence of intestinal organoids offers the possibility to solve the above issues. Indeed, a recent study showed that porcine intestinal organoids (PIOs) are more similar to epithelial tissue than IPEC-J2 and are physiologically closer to in vivo conditions than immortalized cell lines through comparative transcriptome analysis of PIO epithelial tissue and IPEC-J2 cells [19]. Intestinal organoids are in vitro culture models consisting of multiple intestinal cell types with petal-like structures of the hollow lumen, containing both villi and crypt-like domains, capable of long-term self-renewal, and they can be stably cryopreserved and resuscitated [20]. Human intestinal organoid models have been reported and used in various research applications, which are derived from the crypt or induced by pluripotent stem cells (PSCs) [21,22]. However, the human intestinal crypt is usually derived from diseased tissues, and there is individual variability in tissue origin. Compared with crypt-derived intestinal organoids, PSC-derived intestinal organoids possess more advantages, such as avoidance of tissue origin issues and ethical issues, as well as the possibility of gene editing to generate personalized intestinal organoids. In contrast to human studies, there is a paucity of literature on PIOs. Only a few crypt-derived PIOs have been reported. There are no reports on the generation of PIOs derived from PSCs. Targeted induction of porcine PSCs to generate intestinal organoids seems feasible considering the high similarity between the porcine and human genomes, providing additional insights for studies on the porcine intestine.

In this review, we summarize the research progress of PIOs, highlighting their culturing, establishment and development, and applications in agricultural, veterinary, and biomedical research. We also propose further improvements to the methods used to culture PIOs and future applications.

## 2. Culturing, Establishment and Development of PIOs

In 2007, Barker et al. identified leucine-rich repeat sequence G protein-coupled receptor 5 (Lgr5)-positive cells located at the base of intestinal crypts and found that Lgr5-positive basal columnar cells of the crypts afforded all epithelial lineages within 60 days, confirming that these cells were the stem cells of the small intestine and colon [23]. On the basis of this innovative discovery, in 2009, Clevers et al. successfully established mouse intestinal organoids by culturing mouse Lgr5-positive intestinal stem cells in a specific differentiation medium [24]. In 2011, the same group established crypt-derived human intestinal organoids by adding nicotinamide and various small-molecule inhibitors to the cultures used to promote the growth of mouse organoids [25], while PSC-derived human intestinal organoids were established by Spence et al. in the same year [22]. Subsequently, other researchers reported the successful culturing and establishment of intestinal organoids in cattle, pigs, dogs, cats, chickens, and bats [20,26,27,28,29,30]. Self-renewal and differentiation of intestinal stem cells promote organoid expansion, which is regulated by multiple signaling pathways. The Wnt signaling pathway plays an important role in promoting cell proliferation and self-renewal [31], the Notch pathway contributes to cell differentiation [32], the BMP signaling pathway can inhibit β-catenin protein activity [33] (thus, its inhibition by the antagonist Noggin contributes to stem-cell renewal [34]), and epidermal growth factor (EGF) promotes cell proliferation [35]. Other required complementary factors include B27 supplement, nicotinamide, N2 supplement, *N*-acetylcysteine, Y-27632 (ROCK protein kinase inhibitor), SB202190 (p38 MAPK inhibitor), and A83-01 (TGF-β receptor inhibitor), regulating various signaling pathways to ensure the morphological maintenance and long-term culture of intestinal organoids [36].

**Figure 1:**
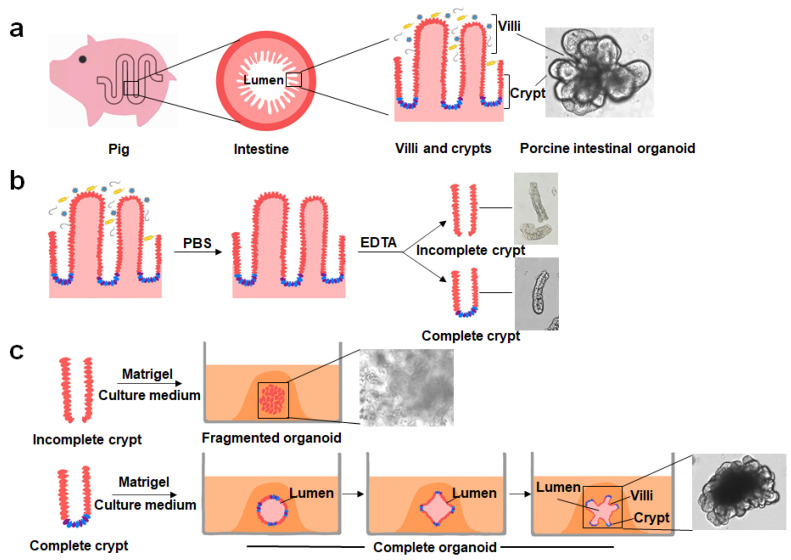
Intestinal structure, crypt location, and organoid formation. (**a**) The physiological and cross-sectional structure of the porcine intestine and the corresponding positions of intestinal villi and crypts in physiological states and porcine intestinal organoids. (**b**,**c**) Structure of complete intestinal crypts containing intestinal stem cells and Paneth cells at the base. The isolated crypts were embedded in Matrigel and then formedpetal-like intestinal organoids by adding various growth factors.

Benefiting from the successful establishment of human intestinal organoids and the elucidation of the signaling pathways associated, Gonzalez et al. for the first time successfully cultured PIOs derived from piglet jejunal tissue in 2013 [27]. Since this work, PIOs derived from different intestinal segments have been established rapidly and applied as in vitro models in various research fields. Two key factors are required for the successful culturing of crypt-derived PIOs: the acquisition of complete porcine intestinal crypts and the appropriate culture system necessary for growth development (Figure 1). The crypts are located in the depression between intestinal villi, and the bottom is arranged by stem cells and Paneth cells in a “U”-shaped structure. The porcine intestine contains many microorganisms and needs to be washed repeatedly with phosphate-buffered saline (PBS), treated with ethylenediaminetetraacetic acid (EDTA) to loosen its structure, and then repeatedly blown with a pipette to obtain a crypt suspension [25]. However, the crypt can easily fragment because of the lack of tissue protection during isolation, resulting in the loss of the underlying structure containing stem cells and Paneth cells and subsequent poor efficiency in cultures. The acquisition of porcine intestinal crypts is based on a previously reported method [37] with slight modifications [38,39,40,41]. Although pigs and humans share a high degree of physiological similarity [11], heterogeneity between them may lead to the fact that the isolated method of human intestinal crypts is not fully applicable to the isolation of porcine intestinal crypts. Previous studies revealed clear variability in the isolation results even when using the same method to isolate different intestinal segments of the crypt in pigs [39], suggesting that a different method is needed for isolating the porcine intestinal crypt. Our recent study showed that complete and many porcine jejunal crypts can be obtained by incubating intestinal tissue with PBS containing 10 mM EDTA and 1 mM dithiothreitol (DTT) on a plate shaker at 70 rpm/min for 25 min at 4 °C, and then vortexed twice for 10 s each with a vortexer at the lowest speed with the capacity of suspending intestinal tissues. This method was also suitable for separating crypts from other intestinal segments.

The development of intestinal crypts into organoids requires the suspension of intestinal crypts in a Matrigel rich in laminin to support three-dimensional (3D) growth and the addition of various factors required to grow intestinal organoids. IntestiCult is a serum-free, commercial culture medium designed specifically for culturing mouse intestinal organoids. Researchers have attempted to use media for culturing mice intestinal organoids to culture PIOs for related studies [38,42,43]. Some studies found that PIOs cultured in IntestiCult displayed a less differentiated organoid morphology [44]. Culturing human intestinal organoids has been standardized and applied in research [45,46], and, on the basis of the similarity between the porcine and human intestines, other researchers have used the medium components from cultured human intestinal organoids to establish PIOs [27,40,41]. However, the resulting PIO structures had few or no emergent structures in the proliferating areas, suggesting a less differentiated organoid pattern. Moreover, the resulting PIOs were not cultured over the long term, as performed for human or mouse intestinal organoids. Previous studies have shown that three proteins, Wnt3a, R-spondin1, and Noggin, play key roles in culturing PIOs [36]. These proteins are commercially available and used to culture PIOs but are expensive and not suitable for some large-scale screening experiments. Thus, establishing cell lines that stably express Wnt3a, R-spondin1, and Noggin and harvesting these three key proteins from cell supernatants is a cost-effective approach. Although cell lines simultaneously expressing all three proteins (L-WRN) or only expressing Wnt3a are available from the American Type Culture Collection (ATCC), the fixed proportion of three proteins derived from L-WRN cell supernatants is insufficient to support the long-term culturing of porcine organoids [29]. This observation indicates that an appropriate ratio among the three proteins is important. In addition, significant differences in the composition of the medium used to culture human and mouse intestinal organoids [22] suggest that different species of intestinal organoids require different cultures. Standardization of intestinal organoid culture techniques is a prerequisite for achieving experimental reproducibility, reducing inter-laboratory variation, and producing high-quality studies. Therefore, it is necessary to explore the conditions for isolating porcine intestinal crypts and organoid cultures to determine an optimal and universally applicable protocol. Our recent study showed that it is important for successful long-term culturing of PIOs to add different ratios of Wnt3a, R-spondin1, and Noggin cell culture supernatants. We also found that the concentration of Wnt3a was critical for the growth of PIOs.

**Figure 2:**
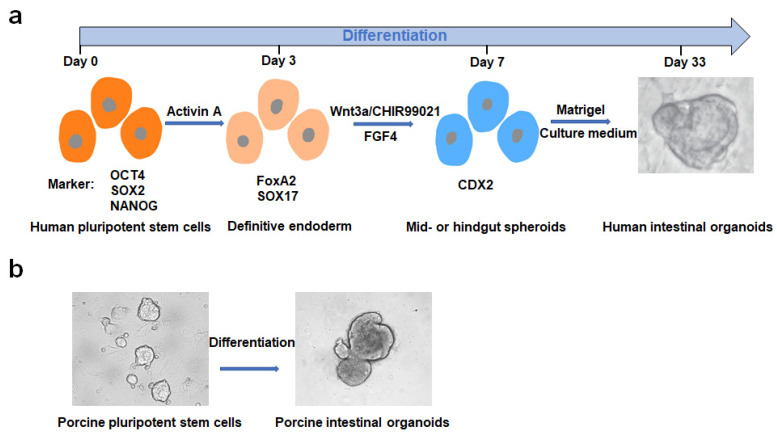
Schematic diagram of a specific method for inducing pluripotent stem cells to form intestinal organoids. (**a**) Pluripotent stem cells expressing pluripotency markers (OCT4, SOX2, and NANOG) are first treated with activin A to form the FoxA2^+^ and SOX17^+^ definitive endoderm. This definitive endoderm is treated with Wnt3a/CHIR99021 and FGF4 to form CDX2^+^ spheroids. The spheroids are embedded in Matrigel and form intestinal organoids with the addition of various growth factors. (**b**) Porcine pluripotent stem cells are cultured in the feeder-free state by adding feeder cell supernatants followed by treatment with activin A, Wnt3a, and FGF4 to form CDX2^+^ spheroids. The formed CDX2^+^ spheroids are then embedded in Matrigel and form intestinal organoids with the addition of various growth factors.

Following the rapid development of PSC technology, targeted induction of human PSCs to form intestinal organoids [21] has considerably widened the method of building organoid models and their application in related fields. In vitro directed differentiation of human PSCs into intestinal organoids mainly includes the following steps (Figure 2): (1) the formation of a definitive endoderm (DE) induced by activin A; (2) the production of the posterior endoderm, hindgut specification, and morphogenesis induced by fibroblast growth factor 4 (FGF4)/Wnt3a; (3) midgut or hindgut encapsulation in Matrigel to promote intestinal growth, morphogenesis, and cell differentiation. The resulting 3D intestinal organoid contains villi-like structures and crypt-like domains with the expression of intestinal stem-cell markers, as well as goblet cells, Paneth cells, and enteroendocrine cells [21]. These 3D intestinal organoids can be used for in vitro mimicry studies of in vivo organs. For example, induced production of colonic-like organoids was used in a SARS-CoV-2 infection study, revealing that the strong induction of chemokines was similar to that observed in SARS-CoV-2-infected patients [47]. Pigs share a high degree of homology with humans; thus, it is feasible to induce porcine PSCs to produce intestinal organoids. However, early established porcine PSCs did not meet the strict pluripotency criteria [48,49]. In 2019, the establishment of true porcine PSCs was first reported in Hong Kong [50], with characteristic long-term stable culturing and application in routine biological experiments, including gene editing [50]. Culturing porcine PSCs requires the presence of feeder cells, which can affect further induction. We briefly maintained a feeder-free culture of porcine PSCs by adding feeder cell supernatant and then successfully induced porcine PSCs into intestinal organoids (Figure 2) by treatment with activator A, FGF4, and Wnt3a, and the resulting intestinal organoids supported infection by an intestinal coronavirus. The successful establishment of porcine PSCs provides an excellent platform for developing more potent and safer therapeutic strategies.

## 3. PIOs as Models for the Study of Intestinal Pathogen–Host Interactions

**Figure 3:**
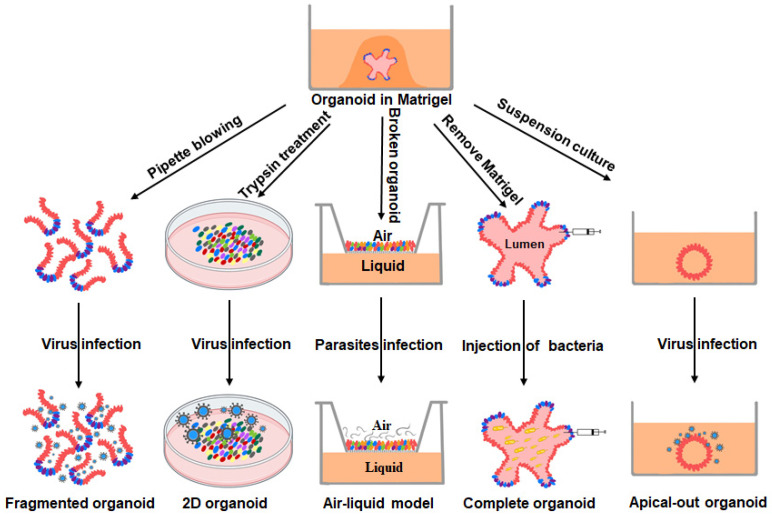
Methods of infecting intestinal organoids with pathogenic microorganisms. The methods of infecting intestinal organoids with pathogenic microorganisms are as follows: (1) blowing apart the intestinal organoids directly to expose the apical membrane and then incubating them with pathogenic microorganisms [45,46]; (2) forming a 2D monolayer by treating the intestinal organoids with trypsin, followed by the addition of pathogenic microorganisms [38,42]; (3) blowing apart the intestinal organoids and then spreading them in a Transwell to form a polarized air–liquid model with multilayer cell accumulation, followed by the addition of pathogenic microorganisms to the air–liquid surface [51]; (4) directly injecting pathogenic microorganisms into the intestinal organoid lumen [52]; (5) exposing the apical membrane inside the intestinal organoid by suspension culturing, followed by infection with pathogenic microorganisms [53].

The intestinal organoid is a 3D structural model with the intestinal hollow lumen in the interior and the exterior encapsulated by Matrigel. The presence of the structure limits the entry of pathogens, which poses a challenge for the study of pathogen–host interactions. To address this problem, researchers have developed several solutions (Figure 3): (1) infection of dissociated organoids; (2) addition to two-dimensional (2D) monolayers; (3) Transwell method; (4) microinjection into the intact organoid; (5) reversal of the intestinal organoids apical membrane. Here, we focus on research progress examining the interactions of the host with enteric coronaviruses, bacteria, and parasites using PIOs as in vitro models.

### 3.1. Host–Viral Interactions

Numerous viruses are present in the porcine intestine, but the application of intestinal organoids has focused primarily on the study of swine enteric coronaviruses, such as PEDV, transmissible gastroenteritis virus (TGEV), and porcine deltacoronavirus (PDCoV), which are the major cause of lethal watery diarrhea in neonatal pigs and pose a significant threat to the farming industry and public health [54,55,56]. The establishment of PIOs has accelerated the study of intestinal coronaviruses, deepening our understanding of their pathogenic mechanisms. For example, PEDV infects multiple intestinal cells in PIOs (epithelial cells, cup cells, and stem cells) and suppresses early IFN production. Further studies have revealed that the clinical isolate PEDV-JMS replicates better than the laboratory virus strain PEDV-CV777 [38]. Transcriptomic analysis of PIOs from different intestinal segments infected with PDCoV showed that the distinct host aminopeptidase N (APN, a functional receptor for PDCoV [57,58]) expression profile is a determinant for PIO susceptibility to PDCoV rather than IFN levels [42]. In addition, the infection of porcine jejunal-derived organoids with PEDV, TGEV, and PDCoV revealed different host epithelial responses via a parallel comparison of transcriptomics [42]. PEDV and TGEV infections induced similar transcriptional profiles that differed from the transcriptional profile obtained from a PDCoV-infected porcine jejunal-derived organoid. In contrast to PEDV infection, TGEV and PDCoV infections trigger abundant upregulation of antigen-presentation genes and T-cell-recruiting chemokines in PIOs [43]. Currently, reported infections of PIOs by porcine enteric coronaviruses include two approaches: treatment of PIOs with trypsin to produce a compact monolayer with transmembrane resistance [38,39,42] or polarization of PIOs to direct the apical membrane outward [53]. Both methods significantly promote pathogen infection. Nonetheless, the polarization of PIOs appears to be a superior approach for infecting PIOs because it enables virus infection from the apical membrane while maintaining the 3D structure of the PIOs. In conclusion, these studies suggest that PIOs can serve as a powerful model for in vitro studies of virus–host interactions and provide new insights into the causative agents and pathogenic mechanisms.

### 3.2. Host–Bacterial Interactions

The presence of pathogenic bacteria in the intestine of poultry and livestock seriously affects the farming industry. PIOs play an important role in the study of bacterial pathogenesis because PIOs contain lumen and tolerate bacteria for several days without significant tissue damage [59]. *Salmonella* is a common contaminant in poultry and livestock and is usually carried asymptomatically in the gastrointestinal tract of animals [60]. A previous study confirmed that *Salmonella* species are highly susceptible to crypt-derived PIOs [20]. Similar PIOs have also been used in studies of *E. coli*, an enteric pathogen that causes post-weaning diarrhea in piglets [61]. Under the stimulation of enterotoxins secreted by *E. coli*, the porcine intestinal compartment exhibits swelling, as well as electrolyte and water imbalance, and it secretes inflammatory markers [44]; further studies have shown that toxin-producing *E. coli* inhibits intestinal stem-cell expansion and disrupts the integrity of the intestinal mucosa through downregulation of the Wnt/β-catenin signaling pathway [62]. A similar phenotype was reported for PIOs treated with deoxynivalenol, a toxin produced by mycobacteria [63]. Bacteria-derived cholera toxin treatment of PIOs causes typical signs of cholera toxin poisoning, which is characterized by increased short-circuit currents and increased epithelial chloride levels [64]. In addition to being a model for in vitro studies of pathogenic bacteria, the intestinal organoid is also a model for in vitro studies of probiotic bacteria. *E. coli* strain Nissle has been used as a probiotic and therapeutic agent to protect mice from enterohemorrhagic *E. coli* [65]. Treatment with *E. coli* strain Nissle prevented loss of epithelial barrier function and E-calmodulin expression in human intestinal organoids and prevented increased production of reactive oxygen species and apoptosis. PIOs can be used to study intestine and probiotic interactions and to develop nutritionally relevant therapeutic and preventive strategies. Taken together, these studies suggest that PIOs are a suitable model for in vitro studies of bacteria.

### 3.3. Host–Parasitic Interactions

Most animal models or cancer cell lines used early in parasitic disease research do not generalize to naturally occurring infections [66,67]. Organoids have become a powerful tool for studying parasitic infections in vitro. *Toxoplasma gondii* can be transmitted through multiple routes, but ingestion of undercooked meat is an important route for its entry into the host gastrointestinal tract [68,69,70]. In recent years, the prevalence of *Toxoplasma gondii* in pork has decreased significantly because of in-house farming. However, with the increase in organic and free-range farms, this prevalence has increased again [71]. In vitro studies on *Toxoplasma gondii* showed that PIOs are highly susceptible to *Toxoplasma gondii* [20]. Although the use of porcine organoids to study host–parasite interactions is in its infancy, intestinal organoids of other species have also been used in different parasite studies, such as *Cryptosporidium* [72], *Toxoplasma gondii* [73], *Giardia* [74], and helminths [75]. This in vitro model should improve the labor-intensive and technically difficult traditional animal experiments [76] and provide new strategies for preventing and controlling parasitic diseases.

## 4. Other Applications of PIOs

### 4.1. The Study of Intestinal Nutritional Development

Most early studies on intestinal nutrition focused on mouse and human intestinal organoids. For example, mice treated with sodium selenite increased the number of intestinal samples in culture and significantly upregulated intestinal stemness markers [77]. Growth hormone increased the proliferation of intestinal stem cells in mice and upregulated the expression of stemness markers, e.g., Lgr5, whereas treatment of mice with glutamine affected the differentiation potential of intestinal stem cells [78]. 

The use of pigs as a model for human nutrition studies has received more attention recently. A previous study used piglets as a model for studying pediatric nutrition and metabolism [79]. Che et al. used an intrauterine growth restricted pig model to explore the effects of postnatal nutritional restriction on the oxidative status of neonates and confirmed that postnatal nutritional restriction leads to impaired antioxidant defense systems in intrauterine growth-restricted pigs [80]. Feeding pigs with different ratios of fat and fiber affects the gut microbiota and microbial metabolites, suggesting that pigs are a promising model system for studying the interaction of the human diet with intestinal microbiota [81]. The successful cultivation of PIOs can reduce the use of animal experiments and provide valuable or suitable alternatives for human intestinal development. Weaning stress in piglets usually damages the intestinal stem cells of piglets, causing diarrhea and leading to great economic losses to the pig industry [82]. The addition of vitamin A during feeding can effectively alleviate weaning stress in piglets, and in vitro experiments have shown that vitamin A can significantly change the morphology of intestinal organoids [83]. Another study indicated that treatment with glutamine enhanced proliferation and renewal of porcine jejunal crypts [84], confirming that PIOs are a promising alternative model for in vivo intestinal growth and development studies.

### 4.2. Drug Discovery

Inappropriate models for preclinical drug experiments may lead to the failure of clinical trials. Several conventional biological experimental techniques can be used on organoids, such as RT-qPCR, Western blot, and CRISPR/Cas9 [46,85,86,87], thereby accelerating the application of organoids in drug discovery. Different human organoids have been applied to drug discovery. For example, a previous study confirmed that the chemotherapeutic drug cisplatin exhibits toxicity toward kidney organoids in a dose-dependent manner [88], while different drug treatments caused alterations in the beat rates of a heart organoid [89]. In addition, liver organoids enabled sensitive assessment of acetaminophen-related toxicity [90]. Colonic-like organoids supported the high-throughput screening of Food and Drug Administration (FDA)-approved drugs against SARS-CoV-2 infection, and several effective drugs were identified, including imatinib, mycophenolic acid, and quinacrine dihydrochloride [47]. However, human organoids are usually derived from diseased tissues or generated by inducing PSCs. Although organoids developed from diseased tissues can generate human drug screening models for specific diseases, this choice of organoid may affect research outcomes because of the large inter-individual variability. In addition, the generation of human intestinal tissue derived from PSCs in vitro usually takes a long time [21]. As a species with close genetic homology and organ anatomical and physiological similarities to humans, porcine organoids can be obtained from healthy tissues, which avoids the impact of tissue origin differences on the stability of experimental results. Our recent study showed that porcine PSCs can be targeted to produce intestinal organoids, which exhibit great potential for various applications and avoid the ethical problems associated with using human PSCs. Other researchers used porcine, monkey, and human colonic organoids to test the toxic responses of anticancer drugs irinotecan and regorafenib and found that porcine colonic organoids were closer to human colonic organs than monkey colonic organs [41]. Although using porcine organoids for drug screening is still in its infancy, their use has significant potential, especially in establishing porcine PSC-derived organoids.

### 4.3. Gene Editing

Inactivating mutations in human motor molecule myosin Vb (MYO5B) cause microvillous inclusion body disease (MVID), a congenital diarrheal disease caused by genetic mutations [91,92,93]. During the first week of life, life-threatening diarrhea requires early treatment with total parenteral nutrition [94]. There is no definitive treatment for MVID. Researchers developed a porcine MVID model by gene editing to study the pathogenesis of human MVID and establish PIOs for in vitro simulation [95]. This study showed that the porcine MVID model was very similar to human MVID. The application of gene-edited pigs avoided ethical issues, and the generation of porcine somatic organoids with targeted gene editing provided important inspirations for future studies.

The generation of personalized organoids by gene editing is a future research direction. Although it is possible to study the function of a particular gene by transferring the gene into a human intestinal organoid [96] or by generating a model animal with gene editing, both genetic manipulations at the organoid level and the generation of model animals are typically complex and time-consuming. PSCs can develop into model animals in vivo after being genetically edited in vitro, and recent studies have shown that PSCs can be directly induced to generate organoids in vitro [22,47,88]. This result suggests that it is feasible to generate personalized porcine organoids through in vitro induction of gene-edited porcine PSCs, which may replace the use of related model animals. 

## 5. Concluding Remarks and Future Prospects

The establishment and use of PIOs have facilitated the progress of many key research areas in recent years. In this review, we summarized the recent progress in establishing PIOs and their use as in vitro models in the study of intestinal pathogen–host interactions, nutritional development, drug discovery, and gene editing potential. The structural and genetic similarities between the porcine and human intestines provide an alternative model for human intestinal development, disease research, and drug screening. However, several problems remain to be solved. Firstly, the currently reported PIOs are not amenable to long-term passaging of cultures, which may be caused by the inadequacy of the medium composition. Secondly, many studies have used intestinal cell monolayers from single-cell suspensions of enzymatically dissociated porcine organoids to perform pathogenic infection research; however, this method loses the advantage of the 3D structure of the organoids. Although polarity reversal can maintain the 3D structure of organoids [53], this method remains discrepant when used to simulate in vivo conditions. Thirdly, PIOs do not contain structures such as surrounding tissues, immune cells, and blood vessels during in vitro culturing, which may differ from the real situation in vivo. Thus, future challenges include increasing the complexity of porcine intestinal models. Lastly, PIO cultures require Matrigel to provide 3D supports, but Matrigel is derived from mouse sarcomas, and the complexity and uncertainty of the composition may have implications for transplantation-related studies. The use of hydrogels or synthetic scaffolds with a more defined composition as 3D supports represents a future option.

The emergence of new technologies, materials, and methods will broaden the application of PIOs. Currently, human intestinal organoids can be induced and differentiated from long-term cultured PSCs and applied to related research [21,47], which will fundamentally address the source variability of organoids and greatly enhance the reproducibility of experiments. The successful culture of porcine PSCs offers the possibility of inducing differentiation of stem cells into intestinal organoids [50], which can be genetically edited, and the edited stem cells can be induced to form more customized organoids. In conclusion, continuing research on PIOs will lead to PIOs becoming a powerful tool in future research endeavors.

## Data Availability

The data presented in this study are available in the article.
